# Exhaustion of the CD8^+^ T Cell Compartment in Patients with Mutations in Phosphoinositide 3-Kinase Delta

**DOI:** 10.3389/fimmu.2018.00446

**Published:** 2018-03-07

**Authors:** Marjolein W. J. Wentink, Yvonne M. Mueller, Virgil A. S. H. Dalm, Gertjan J. Driessen, P. Martin van Hagen, Joris M. van Montfrans, Mirjam van der Burg, Peter D. Katsikis

**Affiliations:** ^1^Department of Immunology, Erasmus MC, University Medical Center, Rotterdam, Netherlands; ^2^Department of Internal Medicine – Division of Clinical Immunology, Erasmus MC, University Medical Center, Rotterdam, Netherlands; ^3^Division of Pediatrics, Juliana Children’s Hospital, Haga Teaching Hospital, The Hague, Netherlands; ^4^Division of Pediatric Infectious Disease and Immunology, Erasmus MC, University Medical Center, Rotterdam, Netherlands; ^5^Division of Pediatrics, Pediatric Immunology and Infectious Disease, Wilhelmina Children’s Hospital, University Medical Centre Utrecht, Utrecht, Netherlands

**Keywords:** activated phosphoinositide 3-kinase delta syndrome, p110δ, PI3K, CD8^+^ T cells, exhaustion, programmed death receptor-1, checkpoint inhibition

## Abstract

Pathogenic gain-of-function mutations in the gene encoding phosphoinositide 3-kinase delta (PI3Kδ) cause activated PI3Kδ syndrome (APDS), a disease characterized by humoral immunodeficiency, lymphadenopathy, and an inability to control persistent viral infections including Epstein–Barr virus (EBV) and cytomegalovirus (CMV) infections. Understanding the mechanisms leading to impaired immune response is important to optimally treat APDS patients. Immunosenescence of CD8^+^ T cells was suggested to contribute to APDS pathogenesis. However, the constitutive activation of T cells in APDS may also result in T cell exhaustion. Therefore, we studied exhaustion of the CD8^+^ T cell compartment in APDS patients and compared them with healthy controls and HIV patients, as a control for exhaustion. The subset distribution of the T cell compartment of APDS patients was comparable with HIV patients with decreased naive CD4^+^ and CD8^+^ T cells and increased effector CD8^+^ T cells. Like in HIV^+^ patients, expression of activation markers and inhibitory receptors CD160, CD244, and programmed death receptor (PD)-1 on CD8^+^ T cells was increased in APDS patients, indicating exhaustion. EBV-specific CD8^+^ T cells from APDS patients exhibited an exhausted phenotype that resembled HIV-specific CD8^+^ T cells in terms of inhibitory receptor expression. Inhibition of PD-1 on EBV-specific CD8^+^ T cells from APDS patients enhanced *in vitro* proliferation and effector cytokine production. Based on these results, we conclude that total and EBV-specific CD8^+^ T cells from APDS patients are characterized by T cell exhaustion. Furthermore, PD-1 checkpoint inhibition may provide a possible therapeutic approach to support the immune system of APDS patients to control EBV and CMV.

## Introduction

The phosphoinositide 3-kinase–AKT (PI3K–AKT) signaling pathway is involved in many crucial cellular processes including regulation of metabolism, proliferation, apoptosis, cell cycle regulation, and protein synthesis (1–3). In human lymphocytes, the phosphoinositide 3-kinase delta (PI3Kδ) isoform, a heterodimer consisting of the catalytic subunit p110δ (encoded by *PIK3CD*) and regulatory subunit p85α (encoded by *PIK3R1*), is essential for both B cell and T cell development and maturation (4–7). For CD8^+^ T cells, PI3Kδ has been shown to be essential for optimal immune responses to pathogens (8, 9).

Over the past years, patients with gain-of-function (GOF) mutations in PI3Kδ have been described (10–12). These patients suffer from a specific form of primary immune deficiency called activated PI3Kδ syndrome (APDS) (13, 14). This disease is characterized by disturbed humoral immunity resulting in hypogammaglobulinemia, recurrent respiratory tract infections an absent response to polysaccharide vaccination, pulmonary damage, lymphadenopathy, hepatosplenomegaly, an increased risk for hematological malignancies, and an inability to control persistent viral infections such as Epstein–Barr virus (EBV) and cytomegalovirus (CMV) infections (13, 14). Immunophenotypically, these patients have decreased numbers of total CD4^+^ and especially naive CD4^+^ T cells together with increased CD8^+^ effector T cells. Furthermore, they have a relative increase in their transitional B cells accompanied by reduced memory B cells (15). Several studies indicated that the effector function of their T cells is defective, causing an inability to control chronic viral infections including CMV and EBV infections (13, 14).

Impaired T cell effector function can be caused by different mechanisms, one of which is senescence (16, 17). Hallmarks of senescence are permanent cell cycle arrest (18, 19) and resistance to apoptosis (20, 21). Importantly, senescent T cells are metabolically and functionally active and retain their cytotoxic functions and ability to produce and secrete cytokines (22, 23). Reduced telomere length and surface-expression of CD57 were used to define senescent T cells. However, to reliably distinguish senescence from other causes of T cell impairment additional markers like senescence-associated β-galactosidase and cyclin-dependent kinase inhibitor 2A (p16Ink4A) can be used (24, 25). Although senescence is age dependent, other factors such as CMV infection can contribute to senescence (26).

Exhaustion of T cells due to chronic antigenic stimulation is another mechanism leading to impaired T cell effector functions. T cell exhaustion was first described in chronic viral infections such as lymphocytic choriomeningitis virus infection in mice (27–29) but is also recognized as an underlying mechanism in immunological failure in human viral infections including HIV infection (30–33) and tumors (34–36). Exhaustion is a hierarchical process (37, 38) by which CD8^+^ T cells first lose their proliferative capacity and IL-2 secretion, followed by diminished secretion of effector cytokines such as tumor necrosis factor (TNF) α and interferon (IFN) γ and eventually they become sensitive to apoptosis, which leads to the loss of these cells (29, 38, 39). Simultaneously, these cells upregulate several inhibitory receptors including programmed death receptor (PD)-1, CD160, and CD244 which, when co-expressed, indicate later stages of exhaustion (40–43). These inhibitory receptors are considered to play a central role in exhaustion. Blocking these inhibitory receptors on exhausted CD8^+^ T cells can restore or improve their function in chronic viral infections as shown *in vitro* (41, 42, 44–46) and *in vivo* (40, 47–51). In addition, inhibitory receptor blockade was introduced into the clinic to re-activate exhausted T cells in cancer (52, 53).

Previously, total and virus-specific CD8^+^ T cells in APDS patients were shown to have upregulated CD57 expression and reduced proliferative capacity. These findings were interpreted as T cell senescence (12, 54–56). However, patients’ lymphocytes also exhibited an increased rate of apoptosis compared with healthy controls (11, 15, 54), which is not in line with the resistance to apoptosis that has been ascribed to T cell senescence (21). Increased apoptosis sensitivity is associated with exhaustion rather than senescence (39, 57). In addition, APDS patient T cells have been reported to express more PD-1, a receptor associated with T cell activation and exhaustion (12, 54, 55). The PI3Kδ pathway is critical for TCR signaling in CD8^+^ T cells (58) and chronic antigen stimulation alone is sufficient to lead to CD8^+^ T cell exhaustion (59). This raises the question whether GOF mutations in PI3Kδ lead to changes in the activation of T cells which might predispose for T cell exhaustion rather than or in addition to immune senescence. Understanding the mechanisms leading to impaired immune response in APDS patients is a requirement to define the best treatment options for these patients that can support the control of viral infections, which could in turn reduce virus-related morbidities in these patients.

To elucidate the role of exhaustion in APDS patients, total CD8^+^ T cells and CD4^+^ T cells from APDS patients were phenotypically characterized and compared with T cells from healthy individuals and HIV-infected patients (HIV^+^ patients). We have included peripheral blood mononuclear cells (PBMC) from HIV-infected patients since it is well established that HIV infection leads to exhaustion of HIV-specific CD8^+^ T cells but not CMV-specific CD8^+^ T cells in HIV-infected patients (39, 41) and can therefore serve as a positive control for exhaustion. Furthermore, virus-specific CD8^+^ T cells in all three groups were characterized, and the effect of PD-1 blockade on proliferation and effector functions was investigated. Our findings indicate that indeed CD8^+^ T cells from APDS patients are more similar to the ones from HIV^+^ patients and exhibit characteristics of exhaustion. Importantly, we show that blocking PD-1 signaling can increase virus-specific CD8^+^ T cell proliferation and cytokine production. Our findings suggest that CD8^+^ T cells in APDS patients undergo exhaustion, and that this may contribute to the impaired control of persistent viral infections such as EBV and CMV. These findings raise the possibility of checkpoint inhibition as a treatment strategy to support APDS patients to control recurrent or chronic viral infections.

## Materials and Methods

### Cell Samples and Ethical Approval

This study was carried out in accordance with the recommendations of Erasmus MC Medical Ethics Committee with written informed consent from all subjects. All subjects gave written informed consent in accordance with the Declaration of Helsinki. The protocol was approved by the Erasmus MC Medical Ethics Committee.

Ten APDS patients were included with a median age of 27 years (range 6–44 years), and a gender ratio of six males to four females. Nine of the patients have a mutation in the PIK3CD gene (seven patients: E1021K mutation, one patient: S312R mutation, and one patient: R929C mutation) (15), and one patient has a mutation in the PIK3R1 gene (N564K). From the patients including in this study, 9/10 received immunoglobulin substitution therapy, and 4/10 received prophylactic antibiotics. None of the patients received steroids or immune modulating drugs at the time of sampling. None of the APDS patients had active EBV or CMV infection at the time of sampling. Three of the APDS patients are EBV-antibody positive, and two are EBV-antibody negative, for the other patients the EBV status is not known. CMV status is not known from these APDS patients. From the healthy controls, nine are EBV positive. The five HIV-infected patients included have a median age of 42 years (range 35–46), gender ratio is one female to four males, three patients have undetectable viral loads (below 20 HIV copies/ml), and two have detectable viral loads (295 and 4,900 copies/ml, respectively). The median CD4 count is 300 cells/μl (range 50–630 cells/μl), and four out of the five patients are on antiretroviral therapy. The 10 healthy control individuals included have a median age of 27 years (range 18–51) and a gender ratio of five males to five females.

Peripheral blood mononuclear cells were isolated from heparinized venous blood by Ficoll-Hypaque (GE Healthcare Life Sciences) density centrifugation, frozen in freezing media [90% fetal bovine serum (FBS)/10% DMSO], and stored in liquid nitrogen until used. Clinical data were provided by treating physicians. Due to availability of material, not all tests could be performed on all samples.

### Flow Cytometric Immunophenotyping

Peripheral blood mononuclear cells were thawed, rested for 30–60 min at 37°C, and stained with previously determined optimal amounts of tetramers and antibodies. For phenotyping of surface antigens, 0.8–1 × 10^6^ cells were washed with Facs wash [FW, Hanks’ buffered saline solution (Corning), 3% fetal bovine serum (Gibco), and 0.02% NaN_3_], stained with tetramer/antibody mix for 30 min at 4°C, washed two times with FW, and fixed with 1% paraformaldehyde. Anti-HLA-A2-PE antibodies (clone BB7.2) were used to identify HLA-A2^+^ donors. Virus-specific CD8^+^ T cells were identified by using APC- or PE-conjugated HLA class I A*0201-β2-microglobulin tetramers loaded with HIV Gag p17 77–85 (SLYNTVATL) peptide, HIV Pol 476–484 (ILKEPVHGV) peptide, EBV peptide (GLCTLVAML), and CMV peptide (NLVPMVATV) (all tetramers were prepared in the lab). The following directly conjugated monoclonal antihuman antibodies were used: CD3-BV421 (clone UCHT1), CD4-BV650 (SK3), CD8-BV786 (RPA-T8), CD45RA-APC-H7 (HI100), CCR7-PE-CF594 and Alexa Fluor 700 (CD197, 150503), PD-1-BV711 (CD279, EH12.1), CD160-Alexa Fluor 488 (BY55), CD244-PE (eBioC1.7, eBioscience), HLA-DR-BV605 (G46-6), CD38-PE-Cy7 (HIT2), CD57-BV605 (NK-1), TNFα-FITC (MAb11, eBioscience), and IFNγ-PECy7 (4S.B3, eBioscience). All antibodies were purchased from BD Biosciences unless otherwise indicated. When AnnexinV-PerCP–Cy5.5 was used to exclude dead cells, 2.5 mM CaCl_2_ was added to all solutions.

Between 1 and 4 × 10^5^ events were collected per sample within 24 h after staining on an LSRFortessa (BD Biosciences, 4 lasers, 18 parameters) and analyzed using FlowJo software (version 9.9.4, Tree Star). Data are represented as frequency within a defined population.

### *In Vitro* Proliferation

To determine proliferative capacity of proliferation-dye-labeled virus-specific CD8^+^ T cells, thawed PBMC were incubated with 0.1 µM of CellTrace Far Red Cell stain (Invitrogen) in PBS for 20 min at 37°C, and free dye was removed by adding RPMI-10% FBS and incubating for 5 min at 37°C. Cells were spun down and resuspended in RPMI 1640 supplemented with 10% heat-inactivated FBS, 2 mM l-glutamine, 100 U/ml penicillin, and 100 µg/ml streptomycin sulfate and added to 24-well plates in a concentration of 1 × 10^6^ PBMC/ml. To inhibit PD-1/PDL-1 interaction, 10 µg/ml of anti-PD-L1 antibody (CD274, MIH1, eBioscience) or isotype control (Mouse IgG1, eBioscience) was added, and cells were incubated for 30 min at 37°C. Virus-specific peptide in a concentration of 1 µg/ml was added then in appropriate wells: Gag peptide (SL9, SLYNTVATL, ANASPEC), Pol peptide (IV9, ILKEPVHGV, ANASPEC), CMV peptide (pp65, NLVPMVATV, ANASPEC), EBV peptide (BMLF1, GLCTLVAML, ANASPEC), EBV peptide pool (PepMix EBV BMLF1, JPT), or media alone (no peptide stimulation). Purified anti-CD28 (1 µg/ml, clone CD28.2, BD Biosciences) and anti-CD49d (1 µg/ml, clone 9F10, BD Biosciences) were added to all wells. Cells were incubated for 5 days at 37°C in a 5% CO_2_ incubator. Cells were harvested on day 5, counted and resuspended in RPMI-10% FBS/1 μg/ml Brefeldin A (GolgiPlug, BD Biosciences)/anti-CD28 (1 μg/ml)/anti-CD49d (1 µg/ml), and incubated for 6 h to determine cytokine production of these cells. For intracellular staining for cytokines, PBMC were first stained for surface antigens, fixed and permeabilized (Cytofix/Cytoperm, BD Bioscience), incubated with the antibodies (see above) for 60 min, washed two times with Perm/Wash Buffer (BD Biosciences), and fixed with 1% paraformaldehyde.

### Statistical Analysis

All data sets were tested for normal distribution using the D’Agostino–Pearson omnibus test. Relative distributions of T cell subsets and comparisons of population frequencies data were analyzed using either the non-parametric Mann–Whitney *U* test or a *T*-test, dependent on whether or not the data were normally distributed (*p* < 0.05 was considered statistically significant). When more than two data sets were analyzed in the same test, the Kruskal–Wallis test was performed, combined with a Dunn’s multiple comparisons test. Correlations were calculated using a Spearman model for correlation, since in the majority of groups at least one population was not normally distributed. Statistics were performed using the GraphPad Prism program (GraphPad Software, San Diego, CA, USA). Populations’ frequencies per group are represented as mean with SEM.

## Results

### Patient Characteristics

We included 10 patients with APDS. From one patient (Pt1), two samples were included, one taken at age 7 years, and one collected at age 25 years. For analysis of total CD4^+^ and CD8^+^ T cells, we only included the adult sample. For analysis of virus-specific CD8^+^ T cells, we included samples from both time-points. Most of the patients have been described before (15). The majority of patients (*n* = 7) carried the previously described E1021K mutation in *PIK3CD* (11, 12), one carried an R929C mutation in *PIK3CD*, and one carried an N564K (15) GOF mutation in *PIK3R1*. One patient carried a missense variant c.935C>G (NM_005026.3) resulting in an amino acid change p.S312C (NP_005017.3) in *PIK3CD*. This latter variant is also found in the general population with a minor allele frequency of around 2% (SNP reference: rs61755420) (60) and can therefore not be classified as disease causing. However, this patient does suffer from antibody deficiency and autoimmunity and increased phosphorylation of AKT in lymphocyte subsets was found (Wentink, unpublished data). Therefore, we decided to study the effect of this variant together with patients with known disease causing mutations. Two of the patients were HLA-A2-positive (including the patient with samples available as a child and an adult) and EBV-specific CD8^+^ T cells were analyzed.

We compared the PBMC from APDS patients to PBMC from 10 healthy controls and 5 HIV^+^ patients.

### The Phenotype of T Cells in APDS Patients Is Distinct from Healthy Controls and Resembles HIV-Infected Patients

**Figure 1:**
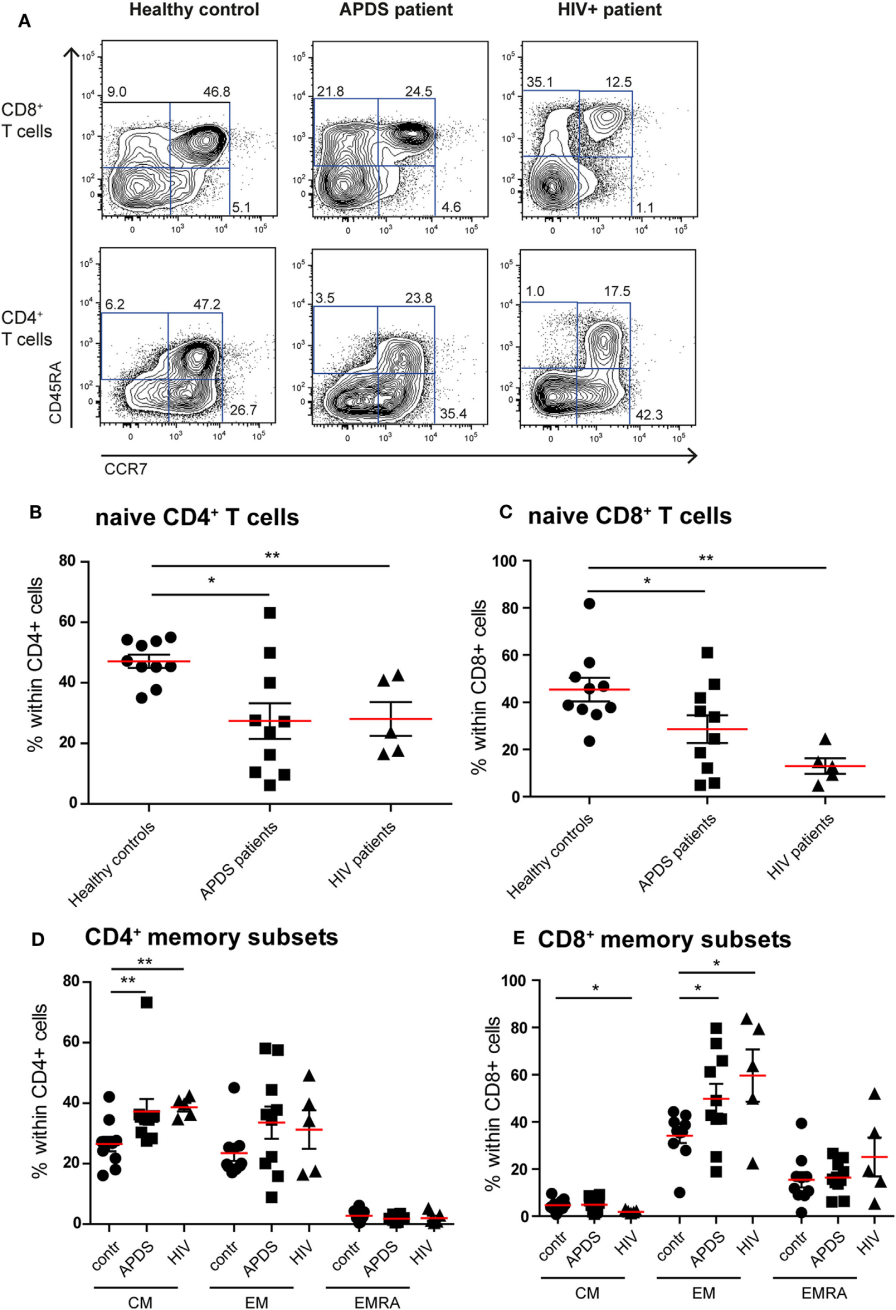
Immunophenotyping of the T-cell compartment of controls (black dots), activated PI3Kδ syndrome (APDS) patients (black squares), and HIV^+^ patients (black triangles). Red lines and brackets indicate the mean and SEM of each group (*p < 0.05 and **p < 0,005). **(A)** Representative dot plots of controls and patients indicating naive (CD45RA^+^CCR7^+^), central memory (CM) (CD45RA^−^CCR7^+^), effector memory (EM) (CD45RA^−^CCR7^−^), and EMRA (CD45RA^+^CCR7^−^) CD8^+^ and CD4^+^ T cell subsets. Numbers depict frequency of cell populations. **(B,C)** The frequency of naive CD4^+^ T cells **(B)** and CD8^+^ T cells **(C)** is reduced in APDS patients and HIV^+^ patients compared with controls. **(D)** The frequency of CM CD4^+^ T cells is increased in APDS patients and HIV^+^ patients. The frequency of the different memory CD4^+^ T cells in controls, APDS patients, and HIV^+^ patients is shown. **(E)** The frequency of EM CD8^+^ T cells is increased in APDS patients and HIV^+^ patients. The different memory CD8^+^ T cell subpopulations are shown for controls, APDS patients and HIV^+^ patients.

Previous studies on the T cell compartment of APDS patients showed a reduction of the naive CD4^+^ and CD8^+^ T cells and an increase in the CD8^+^ T cell effector memory (EM) population (11–13). In our patient cohort, we observed a comparable skewing of the T cell populations. The frequencies of CD45RA^+^CCR7^+^ naive CD4^+^ T cells were significantly decreased in APDS patients compared with healthy controls and comparable with the frequencies in HIV^+^ patients (Figures 1A,B). CD45RA^+^CCR7^+^ naive CD8^+^ T cells were significantly reduced in our APDS patient cohort compared with the healthy controls although not as profound as observed in HIV^+^ patients (Figure 1C). Within memory CD4^+^ T cells, an increase in CD45RA^−^CCR7^+^ central memory (CM) cells was found for the APDS and the HIV^+^ patients compared with healthy controls (Figure 1D). The CD45RA^−^CCR7^−^ EM CD4^+^ T cell frequencies were not significantly different when healthy controls, APDS and HIV^+^ patients were compared (Figure 1D). Frequencies of CM CD8^+^ T cells were comparable between healthy controls and APDS patients as were the CD45RA^+^CCR7^−^ EM re-expressing CD45RA (EMRA) CD8^+^ T cell populations (Figure 1E). A significant increase was found for the CD45RA^−^CCR7^−^ EM CD8^+^ T cell population for APDS patients and HIV^+^ patients when compared with healthy controls. These findings indicate that both patient populations show comparable reduction of naive T cells and increased EM CD8^+^ T cells (Figure 1E).

**Figure 2:**
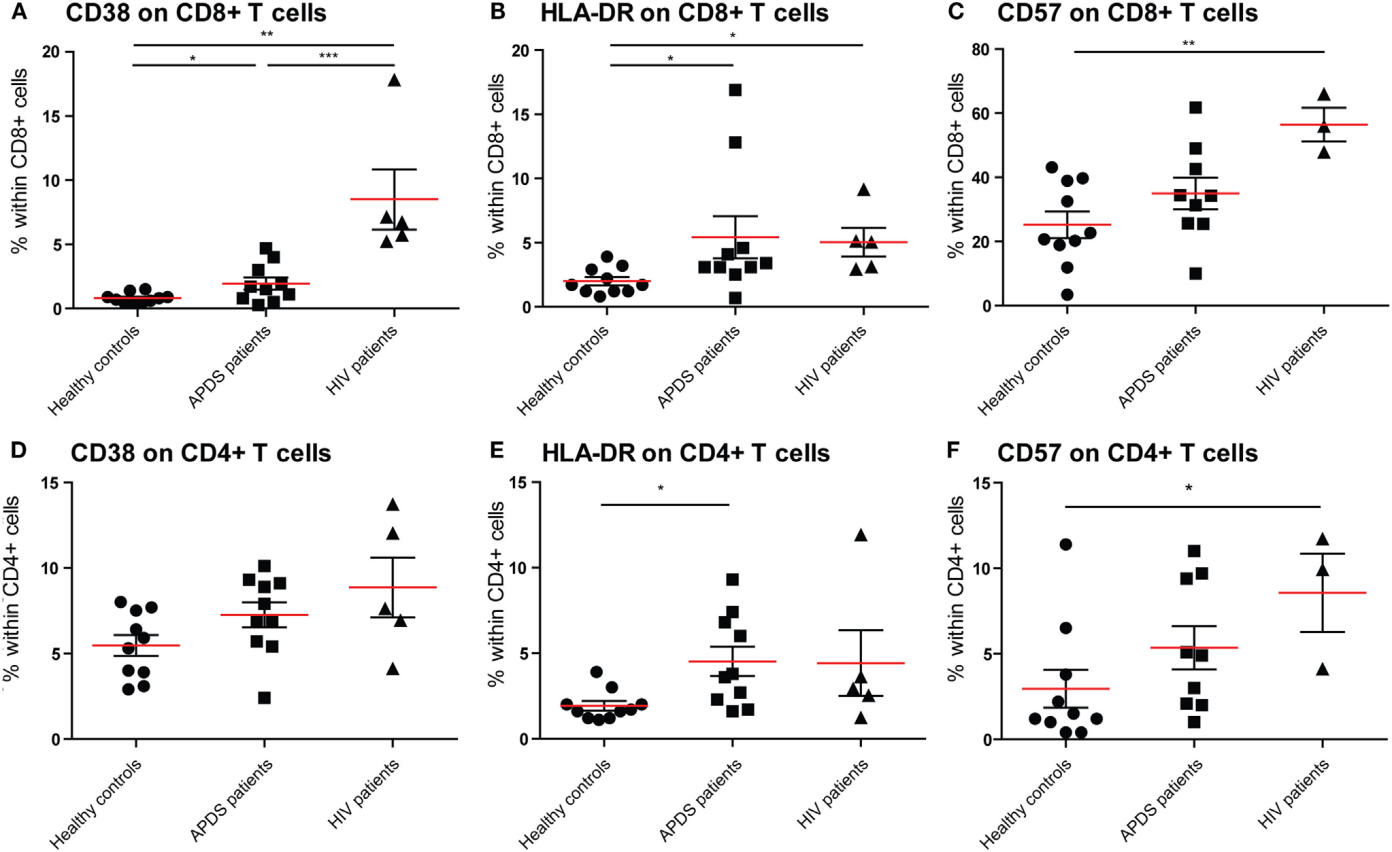
Expression of CD38, HLA-DR, and CD57 on CD8^+^ and CD4^+^ T cells from controls (black dots), activated PI3Kδ syndrome (APDS) patients (black squares), and HIV^+^ patients (black triangles). Red lines and brackets indicate the mean and SEM of each group (*p < 0.05 and **p < 0,005). **(A)** The frequency of CD38^bright^ cells is increased in APDS patients and HIV^+^ patients compared with controls. The frequencies of CD38^bright^ cells within CD8^+^ cells are shown for healthy controls, APDS and HIV^+^ patients. **(B)** The frequency of HLA-DR^+^ cells is increased in APDS patients and HIV^+^ patients compared with controls. Frequencies of HLA-DR^+^ cells within CD8^+^ T cells are shown. **(C)** Frequency of CD57^+^CD8^+^ T cells is not increase in APDS patients. Frequencies of CD57^+^ cells within CD8^+^ cells shown for healthy controls, APDS patients, and HIV^+^ patients. **(D)** Frequency of CD38^+^CD4^+^ T cells is comparable in healthy controls, APDS patients, and HIV^+^ patients. **(E)**, Frequency of HLA-DR^+^CD4^+^ T cells is increased in APDS patients. Frequency of HLA-DR^+^ cells within CD4^+^ T cells shown for healthy controls, APDS patients, and HIV^+^ patients. **(F)** Frequency of CD57^+^CD4^+^ T cells is not increased in APDS patients compared with healthy controls. Frequency of CD57-expressing cells within CD4^+^ T cells shown.

To examine the effect of the GOF mutations on chronic activation we determined the expression of several chronic activation markers on T cells from healthy controls, APDS patients and HIV^+^ patients. The frequency of CD38^bright^CD8^+^ T cells was increased in APDS patients compared with healthy controls; however, HIV^+^ patients had an even higher percentage of CD38^bright^CD8^+^ T cells (Figure 2A). Although the frequency of CD38^bright^CD4^+^ T cells was also higher in APDS patients compared with healthy controls this was not significant (Figure 2D). HLA-DR was examined as a second marker of chronic activation, and indeed a significant increase was found for HIV^+^ patients within the CD8^+^ T cell population. HLA-DR expression was significantly increased on CD8^+^ T cells (Figure 2B) and CD4^+^ T cells (Figure 2E) from the APDS patients compared with healthy controls.

We studied the expression of CD57 on CD8^+^ and CD4^+^ T cells, since this was reported to be increased in a subset of APDS patients. We found that in APDS patients 34.9 ± 5.0% (mean ± SEM) of CD8^+^ T cells express CD57, compared with 25.2 ± 4.0% in healthy controls and 56.4 ± 5.2% in HIV^+^ patients (Figure 2C). This indicates that although CD57^+^CD8^+^ T cells are increased in APDS patients, this is not significantly different from the frequency in healthy controls and lower than the frequency in HIV^+^ patients. A small but non-significant increase of CD57^+^ cells was also found within the CD4^+^ T cell population of APDS patients compared with healthy controls (Figure 2F). Overall, the expression of activation markers and CD57 indicate that T cells from APDS patients tend to be more activated than healthy controls and are therefore more alike T cells from HIV^+^ patients.

### APDS Patients Have Increased Inhibitory Receptor Expression on CD8^+^ T Cells

**Figure 3:**
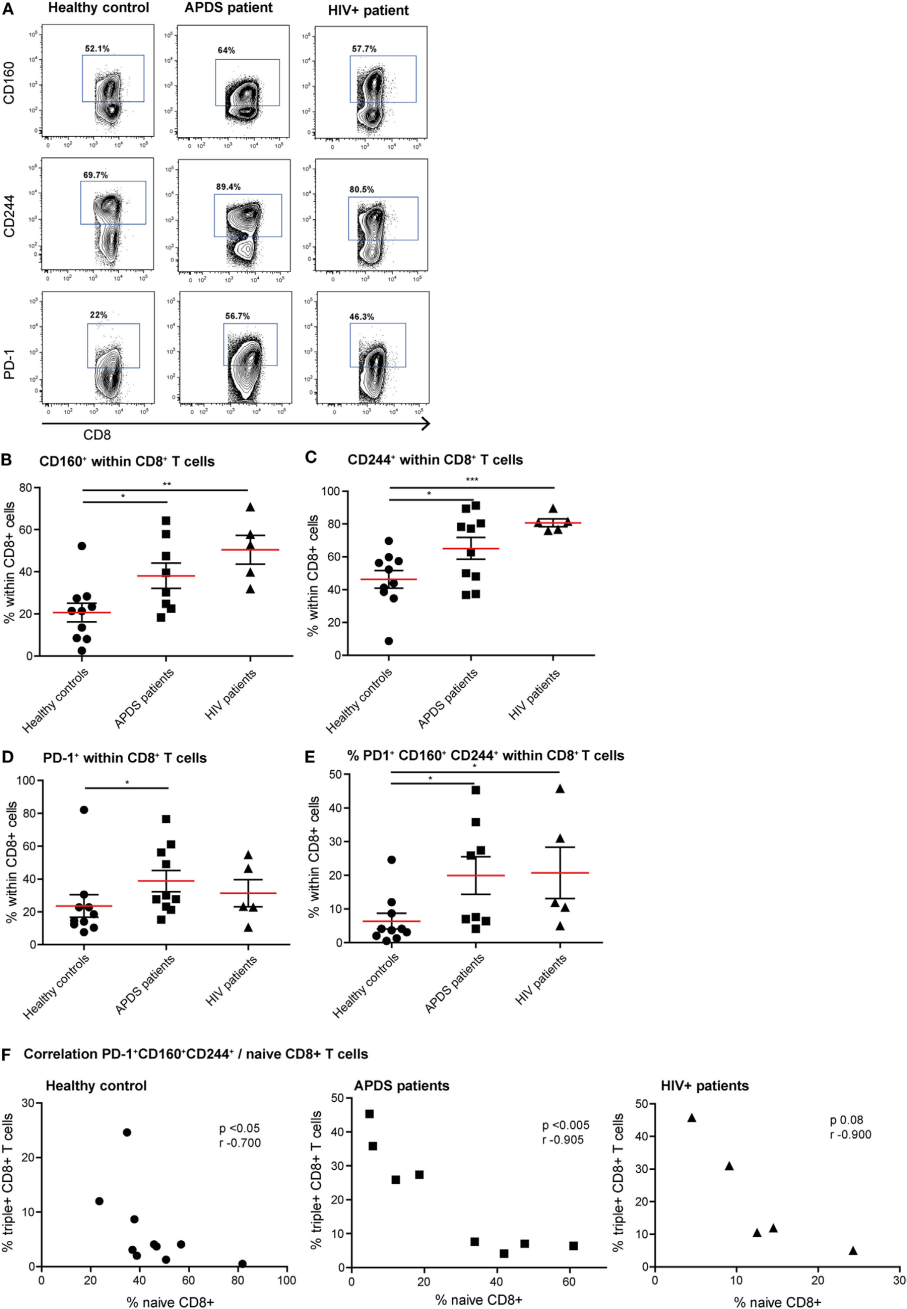
Expression of inhibitory receptors on CD8^+^ cells from controls (black dots), activated PI3Kδ syndrome (APDS) patients (black squares), and HIV^+^ patients (black triangles). Red lines and brackets indicate the mean and SEM of each group (*p < 0.05 and **p < 0,005). **(A)** Representative dot plots of controls and patients indicating populations that were considered positive for inhibitory receptors. **(B)** CD160 expression is increased on CD8^+^ T cells from APDS patients and HIV^+^ patients compared with controls. **(C)** CD244 expression is increased on CD8^+^ T cells from APDS patients and HIV^+^ patients compared with controls. **(D)** Programmed death receptor (PD)-1 expression is increased on CD8^+^ T cells from APDS patients but not HIV^+^ patients compared with controls. **(E)** The frequency of PD-1^+^CD160^+^CD244^+^CD8^+^ T cells is increased in APDS patients and HIV^+^ patients compared with controls. **(F)** The frequency of PD-1^+^CD160^+^CD244^+^CD8^+^ T cells is negatively correlated with the frequency of naive CD8^+^ T cells in controls and APDS patients.

Since exhaustion is a gradual process in which cells over time co-express multiple inhibitory receptors, we studied both the single expression of PD-1, CD160, and CD244 and co-expression of these three receptors on CD8^+^ T cells (Figures 3A–E). As we observed with the activation markers, the expression profile of inhibitory receptors within the APDS patients is very heterogeneous, with some patients in the range of controls and others in the range of HIV^+^ patients. We observed a significantly higher percentage of CD8^+^ T cells from APDS patients expressing CD160 compared with controls (mean 38 ± 6.0 and 21 ± 4.5%, respectively). This frequency in APDS patients was closer to HIV^+^ patients (mean 50 ± 6.8%) (Figure 3B). The frequency of CD244-expressing CD8^+^ T cells within APDS patients and HIV^+^ patients was significantly increased compared with healthy controls (Figure 3C). PD-1 was also found increased on CD8^+^ T cells in APDS patients compared with controls (mean 38.8 ± 6.5 and 23.6 ± 6.9%, respectively) (Figure 3D). Most importantly, we observed a significant increase in the frequency of CD8^+^ T cells expressing all three inhibitory receptors, PD-1, CD244, and CD160 (PD-1^+^CD160^+^CD244^+^), in the APDS patients which was 20 ± 5.6% compared with 6 ± 2.3% PD-1^+^ CD160^+^CD244^+^CD8^+^ T cells in healthy controls. This frequency of PD-1^+^CD160^+^CD244^+^CD8^+^ T cells in APDS patients is comparable with the one observed in HIV^+^ patients (21 ± 7.6%).

We examined whether a history of EBV infection influences the expression of inhibitory receptors on CD8^+^ T cells. From the APDS patients, EBV status information was available from five individuals, with three being EBV-antibody positive and two being EBV-antibody negative. We compared the expression of inhibitory receptors on CD8^+^ T cells from these two groups to the expression of the inhibitory receptors on the CD8^+^ T cells in the nine EBV-positive healthy controls. We found that the frequency of CD160^+^, CD244^+^, PD-1^+^, and CD160^+^CD244^+^PD-1^+^CD8^+^ T cells is highest in the EBV^+^ APDS patients (CD160^+^CD8^+^: 57 ± 4.9%; CD244^+^CD8^+^: 87 ± 3.4%; PD-1^+^CD8^+^: 61 ± 8.2%; CD160^+^CD244^+^PD-1^+^CD8^+^: 36 ± 5.6%), but lower in the EBV^−^ APDS patients and the EBV^+^ healthy controls (CD160^+^CD8^+^: 26 ± 3.8 and 22 ± 4.7%; CD244^+^CD8^+^: 43 ± 5.3 and 48 ± 5.9%; PD-1^+^CD8^+^: 18 ± 3.0 and 25 ± 7.6%; CD160^+^CD244^+^PD-1^+^CD8^+^: 5.6 ± 1.5 and 7.0 ± 2.5% for EBV^−^ APDS patients and EBV^+^ healthy control, respectively). Thus, EBV-antibody positivity is accompanied by increased inhibitory receptor expression on CD8^+^ T cells.

We next analyzed whether reduced naive CD8^+^ T cell frequency and PD-1^+^CD160^+^CD244^+^CD8^+^ T cells correlate in healthy controls and APDS patients. Although a negative correlation was already observed for healthy controls (Figure 3F), the correlation between the frequencies of PD-1^+^CD160^+^CD244^+^CD8^+^ T cells and naive CD8^+^ T cells was highly significant in APDS patients (Figure 3F). For the HIV^+^ patients, this relationship was not significantly correlated. These results indicate that total CD8^+^ T cells from APDS patients have increased co-expression of inhibitory receptors similar to what is observed in HIV^+^ patients. Furthermore, the negative correlation of PD-1^+^CD160^+^CD244^+^CD8^+^ T cells and naive CD8^+^ T cells indicates that exhaustion in this compartment is associated with the skewed subset distribution.

### Virus-Specific CD8^+^ T Cells from APDS Patients Exhibit an Exhaustion Phenotype

**Figure 4:**
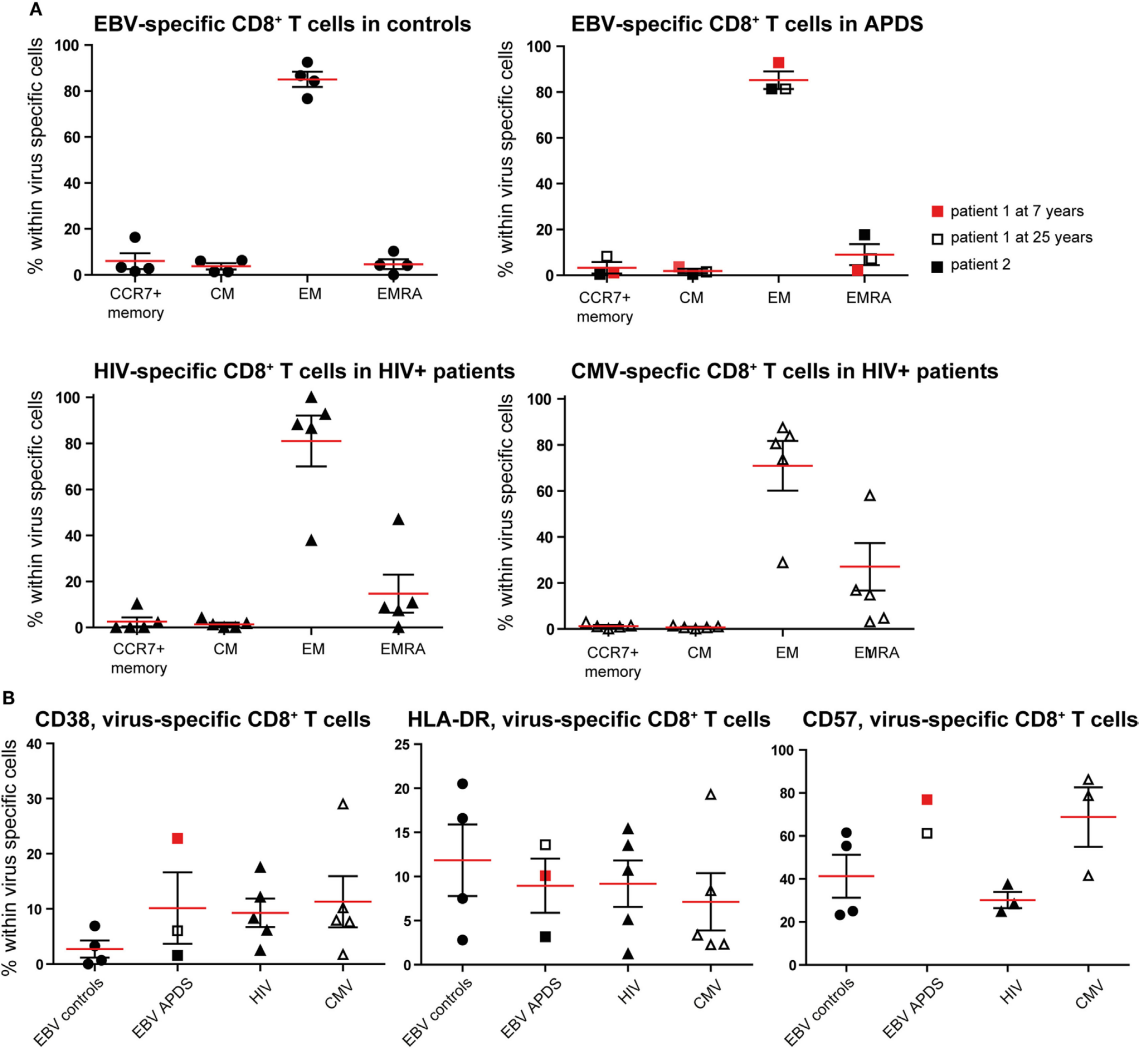
Phenotyping and activation marker expression on Epstein–Barr virus (EBV)-specific CD8^+^ T cells from controls (black dots), EBV-specific CD8^+^ T cells from activated PI3Kδ syndrome (APDS) patient 1 at the age of 7 years (red square), at the age of 25 years (open square), APDS patient 2 (black square), HIV-specific CD8^+^ T cells from HIV^+^ patients (black triangles), and cytomegalovirus (CMV)-specific CD8^+^ T cells from HIV^+^ patients (open triangles). Red lines and brackets indicate the mean and SEM of each group (*p < 0.05 and **p < 0,005). **(A)** Frequency of memory subpopulations [central memory (CM), effector memory (EM), and EMRA, CCR7^+^ memory (CD45RA^+^CCR7^+^)] within virus-specific CD8^+^ T cells shown for healthy controls (EBV-specific CD8^+^ T cells), APDS patients (EBV-specific CD8^+^ T cells), and HIV^+^ patients (HIV- and CMV-specific CD8^+^ T cells). **(B)** Frequency of CD38^+^ (left), HLA-DR^+^ (center), and CD57^+^ (right) cells shown within virus-specific CD8^+^ T cells.

To further compare exhaustion in APDS patients to exhaustion due to HIV infection, we analyzed virus-specific CD8^+^ T cells in both patient groups and healthy controls. HIV-specific CD8^+^ T cells from HIV^+^ patients are highly sensitive to apoptosis, present with a skewed memory phenotype, have proliferative defects, and show increased expression of inhibitory receptors (29, 38, 39). However, in the same HIV^+^ patients, CMV-specific CD8^+^ T cells are not impaired. Using peptide-loaded HLA-A2 tetramers, virus-specific CD8^+^ T cells were analyzed from three APDS patients (EBV-specific CD8^+^ T cells), five HIV^+^ patients (HIV Gag- or Pol-specific CD8^+^ T cells, CMV-specific CD8^+^ T cells), and four healthy controls (EBV-specific CD8^+^ T cells). Within the three APDS samples are two different donors with one donor being represented as a child (7 years, red square in Figure 4) and as an adult (25 years, open square).

The virus-specific CD8^+^ T cells in all four groups have a predominantly CD45RA^−^CCR7^−^ EM phenotype (Figure 4A) which is lowest in CMV-specific CD8^+^ T cells from HIV^+^ patients since these cells have the highest frequency of more differentiated EMRA (Figure 4A). No clear difference was observed for the frequencies of the chronic activation markers CD38 and HLA-DR when the four virus-specific CD8^+^ T cell groups were compared (Figure 4B). All virus-specific cells have higher frequencies of CD38^+^ and HLA-DR^+^ cells than total CD8^+^ T cells (Figures 2A,B) from the same groups. CD57-expressing cells seem to be more abundant within EBV-specific CD8^+^ T cells from APDS patients and CMV-specific CD8^+^ T cells from HIV patients than in EBV-specific CD8^+^ T cells from healthy controls and HIV-specific CD8^+^ T cells from HIV patients.

**Figure 5:**
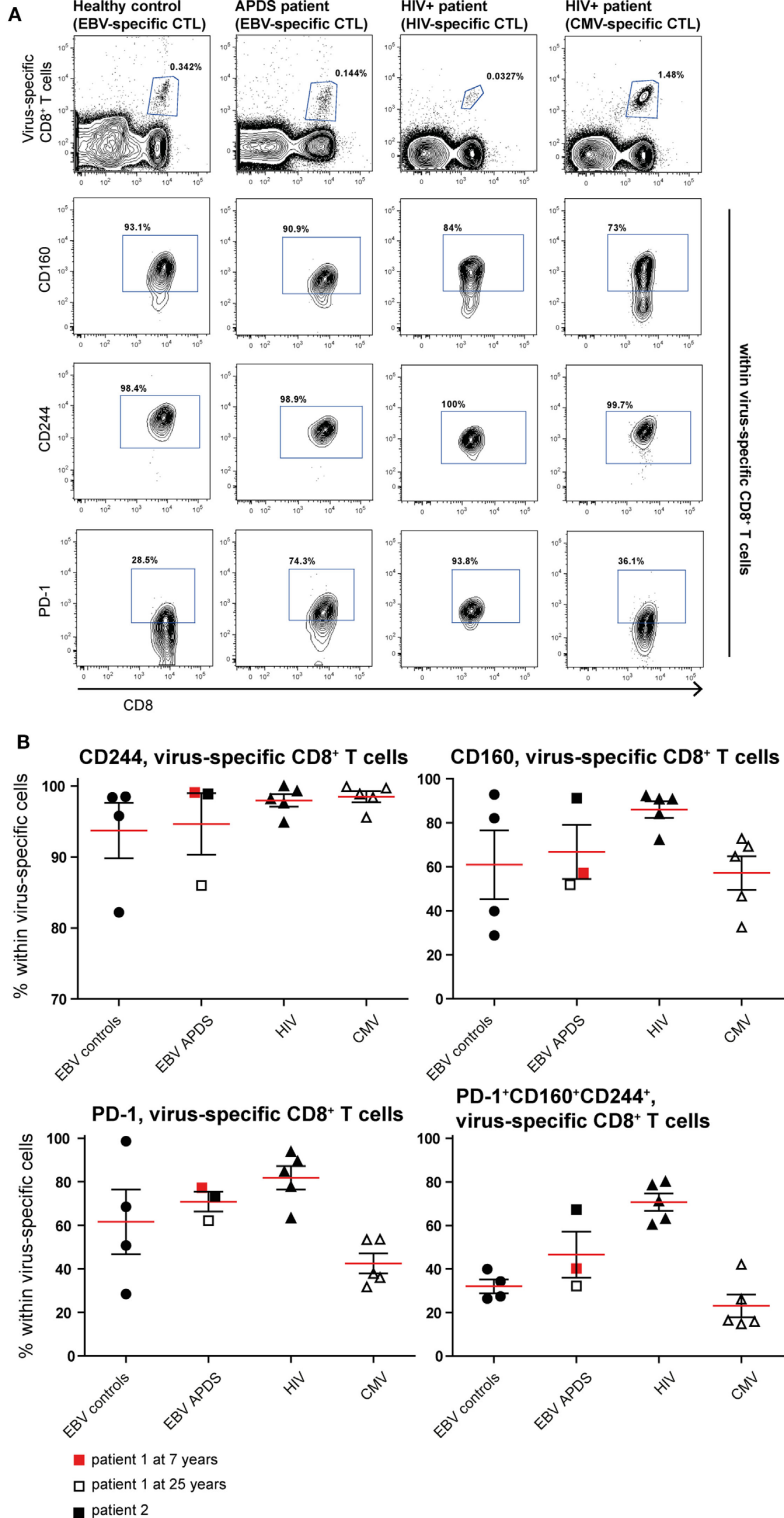
Inhibitory receptor expression on virus-specific CD8^+^ T cells from controls and patients. **(A)** Representative dot plots of healthy controls and patients indicating identification of virus-specific cells and inhibitory receptors positive cells within virus-specific populations. **(B)** Inhibitory receptor expression on Epstein–Barr virus (EBV)-specific cells from controls (black dots), EBV-specific cells from activated PI3Kδ syndrome (APDS) patient 1 at the age of 7 years (red square), at the age of 25 years (open square), APDS patient 2 (black square), HIV-specific cells from HIV^+^ patients (black triangles), and cytomegalovirus (CMV)-specific cells from HIV^+^ patients (open triangles). Red lines and brackets indicate the mean and SEM of each group.

To assess exhaustion in the virus-specific CD8^+^ T cell population, the expression of PD-1, CD160, and CD244 was analyzed (Figure 5). The mean percentage of CD244^+^ CD8^+^ T cells was above 90% in all virus-specific CD8^+^ T cell groups. Frequency of CD160 expression slightly increased on HIV-specific CD8^+^ T cells compared with the other groups. The frequency of PD-1 expressing CD8^+^ T cells was lowest in CMV-specific CD8^+^ T cells from HIV^+^ patients and EBV-specific CD8^+^ T cells from healthy controls but higher within EBV-specific CD8^+^ T cells from APDS patients and HIV-specific CD8^+^ T cells from HIV^+^ patients. We found that within the HIV-specific CD8^+^ T cells 71 ± 4.0% express all three inhibitory receptors, and within the EBV-specific CD8^+^ T cells from APDS patients 47 ± 10.6% express all three inhibitory receptors. EBV-specific cells from controls and CMV-specific cells from HIV^+^ patients have a lower frequency of PD-1^+^CD160^+^CD244^+^ populations (32 ± 3.2 and 23 ± 5.2%, respectively). Compared with overall CD8^+^ T cells, all virus-specific CD8^+^ T cells show increased expression of inhibitory receptors.

### PD-1 Blockade Can Enhance *In Vitro* Proliferation of Virus-Specific Cells in APDS and HIV^+^ Patients

**Figure 6:**
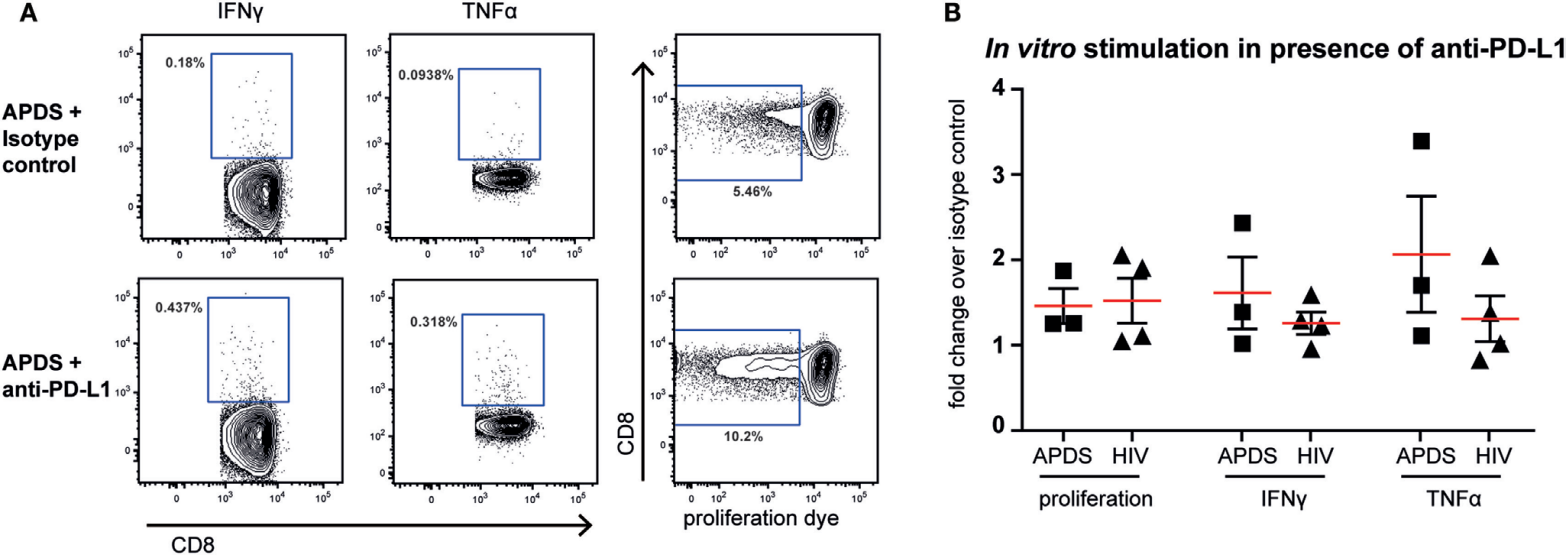
Proliferation and effector cytokine production *in vitro* of Epstein–Barr virus-specific CD8^+^ T cells from activated PI3Kδ syndrome (APDS) patients and HIV-specific CD8^+^ T cells from HIV^+^ patients after peptide stimulation in the presence of anti-programmed death receptor (PD)-L1 antibodies or isotype controls. **(A)** Representative dot plots showing cytokine-producing and proliferated CD8^+^ T cells after 5-day peptide stimulation of peripheral blood mononuclear cells from an APDS patient in the presence of isotype control (upper panel) or anti-PD-L1 monoclonal antibodies (lower panel). Gates indicate cells positive for interferon (IFN) γ, tumor necrosis factor (TNF) α, or cells with diluted cell-trace proliferation dye. **(B)** Anti-PD-L1 antibody treatment resulted in increased proliferation and production of TNFα and IFNγ upon peptide stimulation in both APDS and HIV^+^ patients. Pooled data showing fold change of the frequency of either cells undergoing proliferation or cytokine-producing cells in the presence of anti-PD-L1 antibody over isotype control. Cells were stimulated with viral peptide for 5 days in the presence of monoclonal antibodies. Red lines and brackets indicate the mean and SEM of each group.

Although both senescent and exhausted cells do not proliferate, the mechanism leading to the replicative impairment is different. In senescence, a cell cycle arrest causes the inability of cells to replicate whereas in exhaustion receptor-ligand interaction (such as between the inhibitory receptor PD-1 and its ligand PD-L1) leads to inhibition of TCR-signaling and therefore inhibition of proliferation. Thus, blocking the interaction between receptor and ligand through checkpoint inhibitors could lead to an increase in proliferation of exhausted cells, as shown for HIV-specific CD8^+^ T cells from HIV^+^ patients (44–46) but not in senescent cells. We stimulated PBMC from HIV^+^ patients and APDS patients with virus-specific peptide in the presence of blocking anti-PD-L1 or isotype control antibodies for 5 days before analysis of proliferation and effector function. As reported previously (44–46), inhibition of PD-1/PD-L1 interaction increased proliferation of HIV-specific CD8^+^ T cells in two out of the four tested PBMC samples from HIV^+^ patients by ~2-fold (Figure 6). When PBMC from APDS patients were stimulated with EBV-peptides in the presence of anti-PD-L1 antibodies, proliferation was increased in all three samples compared with samples stimulated with peptide in the presence of an isotype control. Not only did we observe improved proliferation of these cells but also increased effector function as demonstrated by the frequency of cytokine-producing cells (Figure 6). The patient with the highest proliferative and effector cytokine response was also the patient with the highest frequency of PD-1^+^ and PD-1^+^CD160^+^CD244^+^ EBV-specific CD8^+^ T cells. These findings indicate that PD-1 signal inhibition not only increased proliferation but also enabled these cells to release effector cytokines including IFNγ and TNFα. These results suggest that exhausted EBV-specific CD8^+^ T cells from APDS patients can be functionally restored through checkpoint inhibitors, resulting in increased proliferation and effector functions.

## Discussion

How T cell defects due to GOF mutations of PI3Kδ contribute to morbidity in APDS patients is not fully understood. The goal of this study was to determine whether the GOF mutations in PI3Kδ (causing APDS) lead to exhaustion of CD8^+^ T cells. Furthermore, we determined whether virus-specific CD8^+^ T cells against recurrent or persistent infections such as EBV are exhausted in APDS patients and if immune-checkpoint blockade can rejuvenate exhausted CD8^+^ T cells in APDS patients. We compared CD8^+^ T cells from APDS patients to healthy controls and HIV^+^ patients, which served as a control for antigen induced exhaustion of CD8^+^ T cells. Understanding the effect these mutations have on the immune system is central to further treatment strategies to support these patients in controlling chronic infections like EBV and CMV to reduce morbidity and mortality.

In line with previous reports (13, 14), we found significantly reduced frequencies of naive CD4^+^ and CD8^+^ T cells in APDS patients. Loss of naive T cells is thought to be caused by hyperactivation of the mTOR pathway induced by PI3Kδ mutations. This leads to increased glycolysis, proliferation, and differentiation into short-lived effector cells (11, 12). The APDS patients showed a skewed CD8^+^ T cell subset distribution, with increased EM CD8^+^ T cells, which we also found in HIV^+^ patients. Impaired control of viral infection could be an underlying mechanism causing the increased EM pool in HIV^+^ patients. In APDS patients, chronic and/or recurrent viral infections such as EBV and CMV on top of the GOF mutation are likely to contribute to this phenotype. Interestingly, although the EM population is also slightly increased in CD4^+^ T cells, it is the CM population which is significantly higher in both patient groups.

To further examine the effect of PI3Kδ GOF mutations on the total CD4^+^ and CD8^+^ T cell compartments, we analyzed the expression of the chronic activation markers CD38 and HLA-DR. Especially HLA-DR was shown before to correlate with immune activation and disease progression in HIV infection (61). This is the first time that these chronic activation markers are analyzed on T cells from APDS patients. HLA-DR expression was significantly increased on CD8^+^ and CD4^+^ T cells in APDS patients compared with healthy controls, and it is comparable with what is observed in HIV^+^ patients. Our findings indicate that a subpopulation of CD4^+^ and CD8^+^ T cells in APDS patients are chronically activated. However, APDS patients show great heterogeneity, with only a small part of T cells expressing the chronic activation markers, our findings thus suggest that the mutation alone does not lead to a chronic activation of T cells. One exhaustion-inducing factor could perhaps be infection with a persistent virus; we therefore analyzed the expression of CD38 and HLA-DR on CD8^+^ T cells specific for persistent viruses. We did not find significant differences in the expression of activation markers on virus-specific cells between controls, APDS patients, and HIV^+^ patients. Thus, we cannot at this moment conclude that infections with persistent viruses contribute to the higher frequency of HLA-DR^+^ T cells in APDS patients.

Some APDS patients have increased CD57 expression on CD8^+^ T cells, and previously this was interpreted as increased senescence (12, 54, 55). The surface marker CD57 is commonly used as a senescence marker, but T cells in HIV^+^ patients also express increased CD57, indicating that exhaustion and senescence can co-exist in patients (62–64). In our study cohort, the mean CD57^+^ T cell frequency in APDS patients is only slightly increased compared with healthy controls and much lower than that of HIV^+^ patients. However, the CD57 expression in the APDS cohort is very heterogeneous, with some patients comparable with controls and others in the range of HIV^+^ patients. CD57 expression levels were not correlated with age, gender, or mutation in our patient cohort. Neither was this correlated with clinical characteristics such as lymphadenopathy, autoimmunity, malignancies, the expression of other markers we studied here or with B-cell phenotypes. Our results suggest that senescence and exhaustion might not be totally separate processes, but rather two intertwined cellular states that can occur together in specific types of diseases such as APDS.

To further assess exhaustion in APDS patients, we analyzed the expression of the inhibitory receptors CD160, CD244, and PD-1. We have included PBMC from HIV-infected patients since it is well established that HIV infection leads to exhaustion of HIV-specific CD8^+^ T cells but not CMV-specific CD8^+^ T cells in HIV-infected patients (39, 41) and can therefore serve as a positive control for exhaustion. We found that CD244^+^, CD160^+^, and PD-1^+^ CD8^+^ T cells are significantly increased in APDS patients. CD8^+^ T cells expressing all three inhibitory receptors (PD-1^+^CD160^+^CD244^+^), which would indicate the most exhausted state, were also significantly increased in APDS patients and to a similar degree as in HIV-infected individuals. We did not find any correlates for the PD-1^+^CD160^+^CD244^+^CD8^+^ T cell population in APDS patients when age, gender, type of mutations, B cell phenotype, or any other marker we have described were analyzed. When we separated the APDS patients in EBV-antibody positive and EBV-antibody negative, we found that the highest expression of the inhibitory receptors including the concurrent expression of all three inhibitory receptors was observed in EBV^+^ APDS patients. However, EBV^−^ APDS patients had a frequency of inhibitory receptor expressing CD8^+^ T cells which was similar to the one from EBV^+^ healthy controls. These findings suggest that chronic EBV infection or antigen stimulation in APDS patients contributes to the exhaustion of CD8^+^ T cells.

Within virus-specific CD8^+^ T cells, we found increased expression of inhibitory receptors in all subpopulations. Overall, the expression of inhibitory receptors on CD8^+^ T cells from APDS patients has more similarities with HIV^+^ patients, supporting the idea that the PI3Kδ GOF mutations may contribute to exhaustion. This was supported by the highly significant negative correlation between the frequency of naive and PD-1^+^CD160^+^CD244^+^CD8^+^ T cells, indicating that the hyperactivation leading to reduced naive T cells, may also be responsible for the increased expression of inhibitory receptors on T cells. Our findings do not imply that signaling mechanisms leading to exhaustion is identical in APDS and HIV infection although chronic antigen exposure may play an important role in both APDS and HIV patients as has been suggested in mouse studies (59).

Commonly, exhaustion is studied in the context of chronic antigen stimulation due to chronic viral infections and cancer. In APDS, we see a similar increase of exhausted CD8^+^ T cells especially in patients that are EBV-antibody positive implying that chronic antigen stimulation contributes to T cell exhaustion. Chronic TCR stimulation is sufficient to lead to CD8^+^ T cell exhaustion (59). Since PI3Kδ participates in TCR signaling in T cells (58), a cell-intrinsic chronic activation due to the hyperactivity of the PI3K–AKT signaling pathway could be a factor promoting the exhausted phenotype in the absence of specific chronic antigen in APDS. Since not all APDS patients’ T cells show a significant increase in inhibitory receptor expression, the PI3Kδ mutations may be necessary but not sufficient for exhaustion. One mechanism to lower the threshold for exhaustion in APDS could be PI3K-induced epigenetic modifications. Several studies have shown epigenetic alterations in exhausted T cells (65, 66). The altered gene expression patterns induced by epigenetic modifications are rather stable resulting in permanent exhaustion independent of the level of remaining antigen (67, 68). Demethylation of the PD-1 promotor region in exhausted CD8^+^ T cells allows sustained expression of PD-1. That PI3K can indeed alter epigenetic modifications was indicated in a study in mouse embryonic stem cells, suggesting that *de novo* DNA methyltransferases are downregulated due to PI3K-induced AKT, leading to reduced DNA methylation of imprinted loci (69). Therefore PI3Kδ GOF mutations could promote the demethylation of inhibitory receptors or reduce the threshold for such demethylation, thus facilitating the exhaustion in CD8^+^ T cells.

Over the past years, PD-1 blockade has been a subject of research in both HIV and cancer treatment. By blocking inhibitory receptors, exhausted CD8^+^ T cells can regain effectors functions and provide antiviral or anti-tumor immunity. However, upregulation of inhibitory receptors on activated T cells has also a physiological function: they function as negative regulators, downregulating the immune response after successful control of infections (70, 71). They are important to prevent autoimmunity and pathological responses leading to tissue damage. In the absence of inhibitory receptors an increased risk for autoimmunity and immunopathology was reported (72–74). Upregulation of inhibitory receptors in APDS could be a protective mechanism to prevent damage through an overly activated immune system. This hypothesis is supported by a study, which showed how PD-1 signaling can prevent activation of PI3K and AKT phosphorylation, thereby preventing proliferation (75). This benefit of preventing autoimmunity or inflammation in APDS comes at a cost of impairing immunity to viruses. Indeed, we have seen an increase in proliferation and effector cytokine secretion in virus-specific CD8^+^ T cells from APDS patients *in vitro* in the presence of PD-L1 blocking antibodies. The increase of proliferation and cytokine production we have observed in the presence of a PD-L1 blocking antibody is moderate, and this raises the question of biological significance. Since we have observed that the patient with the highest expression of PD-1 showed the highest increase in proliferation and cytokine-producing EBV-specific CD8^+^ T cells this suggests that the more responsive patients to PD-1 blockade will be the highest expressers. To more significantly improve the effector functions in all patients a combination of blocking several checkpoint inhibitors may be required, as indicated by PD-1/CTLA-4 blocking in cancer studies (76, 77) Furthermore, the increase of effector function is similar when EBV-specific CD8^+^ T cells from APDS patients are compared with HIV-specific T cells from HIV-infected patients and within the range reported previously for HIV-specific CD8^+^ T cells (41, 44, 45). Thus, short-term treatment of patients with checkpoint inhibitors during a recurrent EBV and/or CMV infection could be a means to augment efficacy of exhausted virus-specific CD8^+^ T cells and thus reduce EBV and CMV related morbidity. Because inhibitory receptor upregulation may possibly be beneficial in APDS by preventing excessive inflammation or autoimmunity, manipulating this pathway has to be done with caution, and ideally it should be combined with long-term treatment that can reduce the hyperactivation of the pathway, for example selective PI3K inhibition (78).

In summary, we have shown that CD8^+^ T cells from APDS patients have an exhausted phenotype and upregulated expression of inhibitory receptors. This exhaustion of the CD8^+^ T cells contributes to the deficient antiviral capacities of the CD8^+^ T cell compartment. Blocking the PD-1–PD1L interaction could be a target for treatment in these patients during recurrent or persistent viral infections.

## Ethics Statement

This study was carried out in accordance with the recommendations of Erasmus MC Medical Ethics Committee with written informed consent from all subjects. All subjects gave written informed consent in accordance with the Declaration of Helsinki. The protocol was approved by the Erasmus MC Medical Ethics Committee.

## Author Contributions

YM and MW performed experiments and analyzed data. VD, GD, PH, and JM contributed to study design and data analysis. Study was conceived and designed by PK, MB, YM, and MW. Manuscript was written by PK, YM, and MW. All the authors have read and approved the manuscript.

## Conflict of Interest Statement

The authors declare that the research was conducted in the absence of any commercial or financial relationships that could be construed as a potential conflict of interest.
